# COGNITIVE AND PSYCHOSOCIAL OUTCOMES OF DRIVING DIFFICULTIES IN OLDER ADULTS: A 5-YEAR STUDY

**DOI:** 10.1093/geroni/igac059.2284

**Published:** 2022-12-20

**Authors:** Yeji Hwang

**Affiliations:** Drexel University, Philadelphia, Pennsylvania, United States

## Abstract

Many older adults find it difficult to drive a car as they age. However, there are lack of studies on the outcomes of driving difficulties among older adults. The aim of this study was to examine the cognitive and psychosocial outcomes of driving difficulties in older adults. This study was a secondary data analysis using National Social Life, Health, and Aging Project Wave 2 (2010-2011) and 3 (2015-2016). This study followed 1,638 older adults that were of the age 65 and older, who had no difficulties driving a car at Wave 2. Montreal Cognitive Assessment Scale, Center for Epidemiological Studies Depression Scale, Hospital Anxiety and Depression Scale, and Perceived Social Isolation Scale were used. For data analysis, chi square tests, t-tests, and regression analysis were used. After 5 years, 11.1% of people began to have difficulties in driving a car (n=180), and 88.9% of people maintained to have no difficulties driving a car (n=1,441). Compared to people who maintained no difficulties of driving a car over time, people who began to have difficulties had more severe cognitive decline (t=4.59, p< 0.001) and more depressive symptoms over time (t=3.253, p=0.001). Univariate regression analysis also indicated that having difficulties of driving resulted in more severe cognitive decline over time (b=0.137, p< 0.001) and more depressive symptoms over time (b=0.097, p< 0.001). Driving difficulties were not related to anxiety or social isolation. As difficulties in driving are related to poor cognitive and psychological outcomes, healthcare professionals should pay more attention to people who experience driving difficulties.

